# Histopathological parameters reported in microinvasive oral squamous cell carcinoma: a systematic review

**DOI:** 10.4317/medoral.25675

**Published:** 2022-12-24

**Authors:** Cristina Saldivia-Siracusa, Anna Luíza Damaceno Araújo, Wilfredo Alejandro González-Arriagada, Francisco Javier Tejeda Nava, Keith D Hunter, Marcio Ajudarte Lopes, Pablo Agustin Vargas, Alan Roger Santos-Silva

**Affiliations:** 1 DDS. Oral Diagnosis Department, Semiology and Oral Pathology Areas, Piracicaba Dental School, University of Campinas (UNICAMP), Piracicaba, Sao Paulo, Brazil; 2 DDS PhD. Oral Diagnosis Department, Semiology and Oral Pathology Areas, Piracicaba Dental School, University of Campinas (UNICAMP), Piracicaba, Sao Paulo, Brazil; 3 DDS PhD. Oral Pathology Department, Faculty of Dentistry, University of Los Andes, Chile; 4 DDS PhD. Faculty of Stomatology, Autonomous University of San Luis Potosí, San Luis Potosí, México; 5 BDS PhD FRCPath. Liverpool Head and Neck Center, ISMIB, University of Liverpool, Liverpool, UK; 6 DDS PhD. Oral Diagnosis Department, Semiology and Oral Pathology Areas, Piracicaba Dental School, University of Campinas (UNICAMP), Piracicaba, Sao Paulo, Brazil; 7 DDS PhD FRCPath. Oral Diagnosis Department, Semiology and Oral Pathology Areas, Piracicaba Dental School, University of Campinas (UNICAMP), Piracicaba, Sao Paulo, Brazil; 8 DDS PhD. Oral Diagnosis Department, Semiology and Oral Pathology Areas, Piracicaba Dental School, University of Campinas (UNICAMP), Piracicaba, Sao Paulo, Brazil

## Abstract

**Background:**

Microinvasive oral squamous cell carcinoma (OSCCmi) is an incipient stage of oral cancer. Through this systematic review, we aim to assess patterns of histopathological outcomes reported in OSCCmi cases.

**Material and Methods:**

An online search in major databases was performed without period restriction, and 2,024 publications in English, Spanish and Portuguese were obtained. After screening and eligibility, 4 studies were selected. The risk of bias was assessed using Joanna Briggs Institute Critical Appraisal Checklist. A descriptive synthesis was conducted.

**Results:**

All 4 publications included were retrospective, reporting a total of 116 OSCCmi patients, with a male predominance (1.6:1) and a mean age of 55.9 years. The main parameters considered for microinvasion were tumor thickness (TT) (range 4-10mm) and depth of invasion (DOI) (range 0,02-5mm). Definition, cut-off values, and assessment of microscopic features were not standardized. Other relevant measures such as perineural or lymphovascular invasion and pattern of invasive front were barely described, and cytological/architectural characteristics were not discussed.

**Conclusions:**

TT and DOI are currently the primary histopathological criteria used to define OSCCmi. Nonetheless, the outcomes of this systematic review showed the absence of standardized quantitative parameters to render the diagnosis of microinvasive OSCC. Therefore, additional studies aiming to standardize histopathological features to diagnose OSCCmi are paramount.

** Key words:**Microinvasive, microinvasion, oral squamous cell carcinoma, oral cancer, histopathological profile, systematic review.

## Introduction

Oral squamous cell carcinoma (OSCC) is a malignant epithelial neoplasm with a high prevalence that tends to be diagnosed in advanced stages (1). In Brazil, studies show that OSCC is commonly diagnosed at stages III-IV (1,2). It has been widely reported that OSCC stage at the time of diagnosis is strongly associated with crucial prognostic factors, such as survival rates and treatment options, resulting in alterations in quality-of-life and survival (2,3). This suggests early diagnosis of incipient OSCC as a must to reduce mortality and comorbidities associated with this disease.

Microinvasive oral squamous cell carcinoma (OSCCmi) is an early-stage form of OSCC (4). While frankly invasive OSCC tends to be a straightforward diagnosis, there is scarce literature regarding objective definitive criteria for microinvasive squamous cell carcinoma of the oral cavity in contrast to the same entity arising in other localizations of the body (4), such as cervix (5). The usual definition of OSCCmi states microinvasion as “confined to superficial stroma or lamina propria” (6). It has also been defined as “confined to the papillary lamina propria defined by the depth of the rete processes, and superficially invasive if the tumor remains confined to the reticular (deep) lamina propria, not yet involving the submucosal tissues mentioned above” (7). However, as stated concerning other localizations, there are problems in diagnostic precision even though the concept of microinvasion initially seems obvious (8).

Since OSCCmi is understood as an incipient malignant disease, it is expected to be associated with better survival in patients diagnosed with this form of OSCC rather than more deeply invasive tumors. Nonetheless, clinical, and histopathological identification of initial lesions such as OSCCmi represents a real challenge for oral and maxillofacial pathologists, especially as current definitions do not realistically encompass the nuances encountered on microscopic analysis.

With this systematic review, we aim to assess existing evidence regarding the histopathological features of microinvasive oral squamous cell carcinoma (OSCCmi). 

## Material and Methods

- Study Design, protocol, and registration

After an initial exploratory literature review, no similar reviews regarding our topic of interest were identified. Therefore, a systematic review of the literature was carried out based on the Preferred Reporting Items for Systematic Reviews and Meta-Analyses (PRISMA) guidelines (9) and was registered in the International Prospective Register of Systematic Reviews (PROSPERO) database (CRD42022323251). The review question was: “Which are the histopathological criteria used to diagnose micro-invasion on oral squamous cell carcinoma patients?”, and the objective was to identify and document the prevalence of histopathological criteria used to diagnose microinvasive oral squamous cell carcinoma on hematoxylin-eosin (HE) stained, formalin-fixed paraffin-embedded (FFPE) samples.

- Eligibility Criteria

Articles were included if they m*et al*l the following criteria: (a) OSCC with described characteristics on histopathological diagnosis compatible with the definition of microinvasion identified on HE in FFPE tissues according to the AJCC: “confined to superficial stroma or lamina propria”; (b) description of diagnostic criteria on microinvasion; and (c) cross-sectional studies, case-control studies, cohort studies, clinical trials, and case series published in the English, Spanish or Portuguese language.

The exclusion criteria were (a) publications unrelated to the topic of the review; (b) lesions outside of oral and maxillofacial complex; (c) types of publications such as non-human studies (animal or *in vitro* research), reviews, conference papers, letters, book chapters, surveys, news, retracted articles, double publication (keeping only the most recent one) and publications without full-text availability; (d) insufficient or unclear reported data on histopathological analysis; and (e) frankly invasive T1-T2 OSCC tumors.

- Information sources and search strategy

Electronic databases (PubMed, Embase, SCOPUS, Web of Science, LILACS, and Cochrane Library) were selected to perform a search on February 18th, 2022, without period restriction, with the aim of identifying articles potentially relevant to this study.

A manual search was also performed in Google Scholar, ProQuest, and reference lists of included articles to detect any eligible articles that may not have been retrieved by the electronic search strategy. The search strategy is presented in Supplement 1. The search and selection of the articles were carried out by two authors (CSS and ALDA).

- Study selection and data collection process

Following the initial search, two reviewers independently conducted the selection process. Rayyan QCRI was used as a reference manager to exclude duplicates, identify relevant articles according to the reading of title and abstract, perform screening and eligibility of full-text articles congruent with the predefined inclusion/exclusion criteria, as well as recording primary reasons for exclusion. Every step of this process was registered on a flowchart according to the PRISMA guidelines. Disagreements were solved firstly by discussion and then by consulting a third author. Then, data extraction was conducted by the primary researcher and revised by a second author. The selected articles were scrutinized to extract the following main data: author(s), year of publication, country, objective, study design, eligibility criteria, the total number of cases, OSCCmi cases, age (mean), age (range), gender, tumor localization, clinical appearance, histopathological criteria, treatment, follow-up period, survival, recurrence, metastasis, and method chosen for statistical analysis if used. Qualitative and quantitative data was tabulated and processed in Microsoft Excel®.

- Risk of individual bias (quality) assessment

To facilitate the assessment of possible risk of bias for each study, we collected information using The Joanna Briggs Institute Critical Appraisal tool (10). An individual checklist with multiple domains was made describing the procedures undertaken for each study. A judgment of the risk of bias was performed from the extracted information according to the amount of positive or negative domains obtained after evaluation. Subsequently, every investigation was rated by two independent authors as “low”, “moderate” or “high” risk. Studies were considered with a low risk of bias if 0-49% of domains were classified as ‘Yes’; moderate risk was represented by 50-75% ‘Yes’ score and high risk of bias was assigned to studies with 76-100% ‘Yes’ score. Disagreements were solved initially by discussion between the two authors, and then by referring to a third one if needed.

- Data synthesis and statistical analysis

We expected to identify the histopathological profile to diagnose an oral squamous cell carcinoma as “micro-invasive”. All results were interpreted according to the information extracted from the included studies. The level of consistency of obtained data was completely associated with the information available. Common extracted data found was categorized into groups for further comparison and analysis. Specific data from each study was also tabulated and considered for further separate description and discussion if relevant to the aim of our study.

A narrative descriptive synthesis covering the studies' findings is provided.

**Figure 1:**
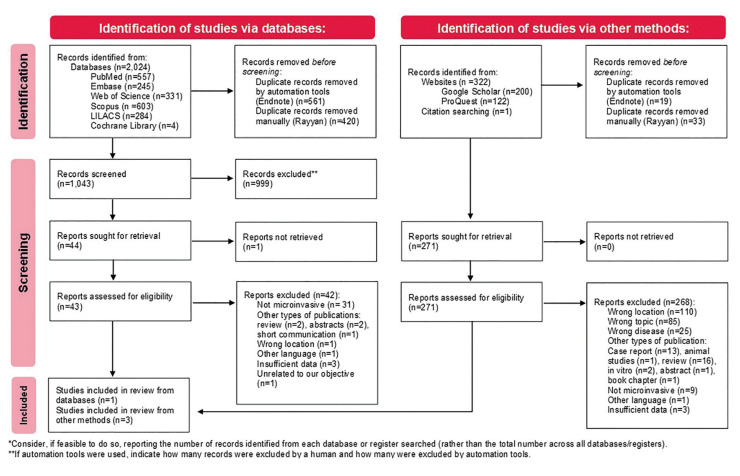
Flowchart describing literature search and overall included studies according to PRISMA guideline (2021 update).

## Results

- Study selection 

A flowchart according to PRISMA guidelines is presented illustrating the selection process (Fig. 1). Using the selected search strategy, 2,024 records published between 1962 and 2022 were initially identified in all databases. 981 duplicate records were excluded, with 1,043 remaining for assessment. After screening by title and abstract, 44 reports were sought for retrieval, and 43 full text articles were assessed for eligibility. Consequently, 42 articles were excluded as they did not meet the eligibility criteria. In addition, 323 records were identified through other methods such as Google Scholar, ProQuest and manual retrieval via reference lists of selected articles. 52 duplicates were removed, and 271 were sought for retrieval and eligibility. Due to incompatibility with the predefined criteria, 268 reports were disqualified. Finally, a total of 4 studies were included (4,6,11,12).

- Description of individual studies

Table 1 summarizes the main clinical and epidemiological data of the included studies. All the papers included were retrospective studies written in English, published between the years 2011 and 2020. Three of the publications were performed in Europe (Italy (6)), United Kingdom (11), and Ireland (12)) and one in Asia (India (4)). Of the four studies included, two of them analyzed clinical features of OSCCmi samples (4,6), one also analyzed histopathological features (4), one aimed to determine a method of DOI measurement (11), and one recorded the frequency of prognostic pathologic features in early OSCC (12). Correlations of obtained results to local recurrence and node positivity were assessed in different studies. The definition of “microinvasion” was diverse.

Altogether, these studies included a total of 408 patients, 116 of which were considered “microinvasive”.

**Table 1:**
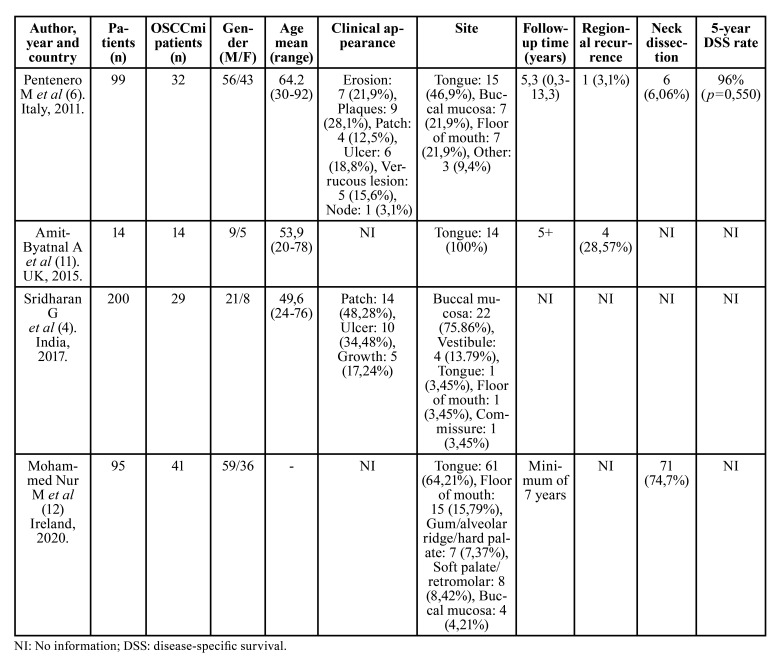
Baseline clinical and epidemiological characteristics of the included studies.

The largest reported population was from India with 200 patients (4); however, the biggest “microinvasive” group belongs to Ireland, with 41 patients (12), followed by Italy (32 patients) (6), India (29 patients) (4) and United Kingdom (14 patients) (11). The mean age was 55.9 years, ranging between 20 to 92 years. One study did not report the average age (12). Regarding gender distribution, a male prevalence was noted. One study did not report gender distribution (4). According to the available information, 145 patients were male and 92 females, resulting in a 1.6:1 male:female ratio. All studies reported localization and the most affected site was tongue (91 lesions), followed by buccal mucosa (33 lesions), floor of mouth (23 lesions), soft palate/retromolar (8 lesions), gum/alveolar ridge/hard palate (7 lesions), vestibule (4 lesions), commissure (1 lesion) and 3 lesions in other unspecified sites. Two studies stated clinical appearance (4,6), with most of the lesions being patches (18 lesions), followed by ulcers (16 lesions), plaques (9 lesions), erosions (7 lesions), verrucous lesions (5 lesions), growths (5 lesions) and a node (1 lesion).

Table 2 summarizes the main histopathological parameters used to diagnose OSCCmi, which were Depth of Invasion (DOI) and Tumor Thickness (TT), with findings reported in 3 (4,11,12) and 2 studies (6,12) respectively. In the 2 studies that used this parameter to determine microinvasion (6,12), OSCCmi lesions ranged in TT from 0.13 to 10mm with a mean thickness of 5.5 mm. Cut-off values used were 4 mm (6) and 10 mm (12). Respecting DOI, lesions ranged from 0.03 to 5 mm with a mean depth of 3 mm. Cut-off values used were 0.5 mm (11) and 5 mm (12). This parameter was also used to determine microinvasion in those studies. No study described nor determined architectural or cytologic findings of the affected epithelium. Parameters such as worst pattern of invasion (WPOI), perineural invasion (PNI), lymphovascular invasion (LVI), differentiation and dysplasia grade were each evaluated in only one article (12).

Surgical excision was the primary treatment modality, reported in 2 studies (6,11). Information regarding regional recurrence was stated in these mentioned studies, with a total of 5 cases of recurrence. Neck dissection was reported in 2 studies (6,12) and performed in 77 patients. Data on survival was evaluated in only one article (6). Follow-up time was heterogeneously stated in 3 studies (6,11,12). One study accompanied their patients for at least 7 years (12), one did it for more than five years (11), and one did it for a mean of 5.3 years (6).

A quantitative synthesis could not be performed as the included studies' present unsuiTable quantitative data and are not sufficiently homogenous in terms of design, variables, and results to conduct a meta-analysis.

- Risk of bias within studies

3 publications were categorized as having an overall moderate risk and 1 as high risk of bias, as clear information was not informed (Table 3). Detailed explanatory information about evaluation of bias risk is available in Supplement 2.

**Table 2:**
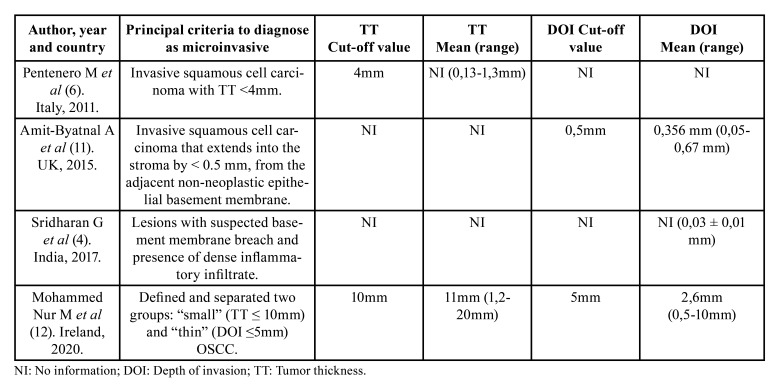
Histopathological parameters used to diagnose OSCCmi on the included studies.

**Table 3:**
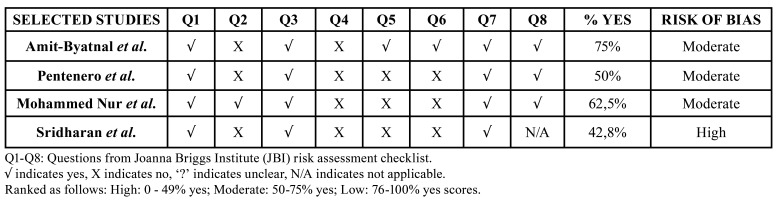
Overall appraisal of risk of bias assessment for the 4 included studies according to The Joanna Briggs Institute Critical Appraisal tool for Cross Sectional Studies.

## Discussion

OSCCmi is an incipient malignant disease of the oral mucosa. Several terms had been proposed to talk about very early presentations of OSCC, like superficially invasive OSCC (13) and small and thin OSCC (12). Microinvasion is considered as beyond the epithelial basement membrane, extending into the superficial adjacent stroma as small nests or islands (14). There are various articles citing the difficulty in diagnosing these micro-invasive tumors in various parts of the body (14,15). At present time, AJCC does not define OSCCmi as a separate entity, opposed to other anatomic regions; in the breast, microinvasive carcinoma is defined as “an invasive carcinoma with no focus measuring >1 mm”, and it has even been stated through this classification that “the clinical impact of multifocal microinvasive disease is not well understood at this time”. This uncertainty could be theoretically extrapolated to the oral cavity.

Besides multiple definitions, histopathological criteria have also been poorly reported. As evidenced by the very small number of studies obtained through this systematic review, there is limited data about OSCCmi, impeding objective analysis of prevalence and incidence. In 2012, Haberland *et al* described clinical and histopathological features of 12 OSCCmi cases and reported them through a conference abstract (13); they obtained an equal sex distribution and an average age of 53 years in this population which presented tumors predominantly in lateral border of the tongue. As a result of this present investigation, we have demonstrated a slight male predominance and a mean age of 56 years. These epidemiological results are consistent with the profile of patients affected by conventional OSCC across the world (1,2,16,17). Furthermore, there are no defined histopathological criteria for identification of microinvasion, and studies assessing microscopic profile or describing findings of this entity are currently minimally recorded and remarkably heterogeneous. Regarding architectural and cytologic features, bulky outgrowth of the epithelial rete pegs and ductal changes were described in 1963 as supportive, but not pathognomonic, histopathological differences in distinguishing carcinoma in situ from micro-invasive carcinoma by Shedd *et al*. (18). Nevertheless, these were never verified in subsequent studies. In our study, analysis of other histopathological characteristics was challenging as there was insufficient and variable data. Grade of dysplasia was reported only in one study (4), and none presented details of the cytologic and/or architectural characteristics of the epithelium. In comparison, cervical microinvasive lesions are studied according to their specific histopathological characteristics, such as nuclear, stroma, and architectural findings (8). As identification of breaches in the basement membrane are helpful, but difficult to identify, previous authors have described the difficulties in early invasion diagnosis to absence of tangible criteria, forcing the professional to rely on judgement and experience (19). This is one of the main reasons why OSCCmi deserves further study (20).

Our results reflect that the microscopic measurement of TT and DOI has been considered a parameter to diagnose OSCCmi. TT considers both exophytic and infiltrative component of the tumor, and it is measured from the highest and most superficial point of the lesion to the deepest point of infiltration (21). In regards of DOI, this measure is usually estimated as the perpendicular distance from the basement membrane region to the deepest point of the tumor front, and it is used to assess the infiltrative component of a malignancy (21). The consensus among pathologists on the maximum dimension in microinvasive OSCCs is limited. We proved this situation for TT and DOI, as designation of cut-off values in each study for both units was merely arbitrary, and no robust evidence was found to support these decisions. Two included studies measured TT (6,12) and three studies used DOI (4,11,12). Also, the variability and frequent lack of clarity in these investigations and in the literature regarding the exact definitions of TT vs DOI is an important issue, particularly when establishing reference points on the epithelium to determine stated dimensions (22). Calculating these measurements is often more theoretical than practical because of the limited thickness of healthy epithelium (22), if any, particularly in incisional biopsies, information which majority of studies do not clarity through their methodology. Localization and tissue disposition would be also a factor to consider because of anatomical variations that could result in a DOI underestimation (23). On this matter, the possible relevance of morphological findings as another factor must be noted in association with TT and DOI; we consider it useful to report differences in atrophic versus exophytic or verrucous lesions, since it is expected that the thickness of these lesions would vary, and therefore this discrepancy could impact the wide ranges found in TT and DOI. Unfortunately, in this review only one article reported 5 verrucous lesions (4) and 5 nodular lesions (6), so the data were unfortunately too sparse to determine their significance. Microinvasion in verrucous lesions is also a topic that has not been widely studied.

Amit-Byatnal *et al* assessed these parameters (TT and DOI) both manually and automatically by an image analysis software and using two different reference points to test variations resulting from these discrepancies, and they attained similar results with non-significant variations (11). Studies have described higher tumor aggressiveness in early lesions with DOI between 3 and 5 mm (12). Considering that the TNM 8th edition staging has taken depth into account by including 5 mm as the cut off between pT1 and pT2 (12) and bearing in mind possible uses of DOI and TT to assess outcomes in patients with OSCCmi, it is extremely relevant to conduct research involving this subpopulation of OSCCmi patients aiming to reach consensus in terms of objective definition and measurements to categorize this disease separately.

Many other features have been associated with predicting a more adverse outcome of OSCC, such as WPOI (24) and PNI (25). However, parameters such as histological differentiation, WPOI, PNI, LVI, and dysplasia were evaluated in just one paper (12). Some studies also highlight possible relevance of lymphocytic or inflammatory stromal response (19,23-25). Heavy inflammatory infiltrate has been described as hampering factor for interpretation of invasion, mostly because it can lead to confusion differentiating between reactive epithelial atypia and oral epithelial dysplasia (26,27) and also because it can hinder basal membrane assessment, resulting a false positive on microinvasion of basal cells. Haberland *et al* reported a moderate to severe lichenoid band-like lymphocytic response in 7 of 12 cases of OSCCmi (13). However, this feature was not analyzed in any of the papers included, and so the relevance of this ascertainment is to be further explored.

Early stages of invasion are critical in terms of diagnosis and prognosis. The obtained data was scarce as expected, since microinvasion has been reported to have a lower incidence of metastatic spread and head and neck surgeons usually do not reoperate these cases. More research would also be interesting to back up this situation.

Finally, it s worth noting the significance of alternative methods that could assist in objective analysis (28), and in this matter, machine learning methods may improve the diagnostic process by the development of artificial intelligence models able to recognize existing patterns imperceptible on routine microscopic evaluation (29).

The limitations of this review must be discussed. First, we experienced difficulties previously reported by Pentenero *et al* (6), because the evidence in the literature regarding “early OSCC” mostly refers as T1/T2 cases; hence, during the eligibility phase of the selection process numerous reports assessed were excluded since the sample would analyze these two groups without distinction. For this reason, it is assumed that incipient microinvasive cases in these samples were not considered on this systematic review as there was no way to extract desired data from the analyses. Also, the divergence in the results of the included studies is not only due to low sampling, but also to methodological differences previously mentioned, such as definitions, diagnostic criteria, and measurements of parameters like DOI and TT, which have been evidenced as critical to diagnose OSCCmi, as well as methods of its detection. As authors consider OSCCmi starting from different principles, there is a tendency to obtain different results even in similar populations or similar objective studies. Consequently, it is expected that the heterogeneity of the included studies could have influenced our results, particularly since data was, in most cases, not comparable.

## Conclusions

OSCCmi is an under-reported incipient malignant entity with a male prevalence that commonly involves tongue and buccal mucosa and has primarily been determined by measures of TT and DOI. Histopathological parameters are not standardized and vary greatly among the evidence available. Characteristics such as cytoarchitectural changes, WPOI, PNI, LVI, and grade of dysplasia have not been considered relevant to the diagnosis of OSCCmi, and there are minimal data about these features and their relation to diagnosis, recurrence, and survival. Thus, there is difficulty in standardizing diagnostic criteria for OSCCmi. This systematic review has highlighted a lack of evidence and absence of agreement concerning histopathological specific parameters to assist proper diagnosis. Consequently, only a few studies were conducted focusing on this population. The significance of a proper histopathological profile assigning defined objective measures such as TT and DOI with distinct established cut-off values is highlighted in this study, as early diagnosed diseases are associated with increased favorable outcomes. By means of this study, we emphasize the need for research concerning this entity. The expansion of this line of research would favor the correct diagnosis of incipient lesions, contributing to a consensus and facilitating microscopic analysis, consequently increasing the number of patients that can be diagnosed prematurely.
